# Analysis of the muscle tissue of Wistar rats submitted to the sciatic nerve compression model and cryotherapy

**DOI:** 10.1590/S1679-45082018AO4206

**Published:** 2018-09-10

**Authors:** Jhenifer Karvat, Camila Mayumi Martin Kakihata, Lizyana Vieira, José Luis da Conceição Silva, Lucinéia de Fátima Chasko Ribeiro, Rose Meire Costa Brancalhão, Gladson Ricardo Flor Bertolini

**Affiliations:** 1 Universidade Estadual do Oeste do Paraná, Cascavel, PR, Brazil

**Keywords:** Muscle, Nerve crush, Cryotherapy, Rats, Wistar, Músculos, Compressão nervosa, Crioterapia, Ratos Wistar

## Abstract

**Objective:**

To evaluate the effects of right sciatic nerve compression and cryotherapy on muscle tissue.

**Methods:**

We used 42 male Wistar rats, subdivided in the following Groups Control, Injury 3, Injury 8 and Injury 15 submitted to nerve compression and euthanized in the 3^rd^, 8^th^ and 15^th^ day after surgery. The Cryotherapy Injury 3 was entailed treatment with cryotherapy by immersion of the animal in recipient for 20 minutes during 1 day, then animals were euthanized at the 3^rd^ day after surgery, and the Cryotherapy Injury 8 and the Cryotherapy Injury 15 was treated for 6 days, and euthanized at the 8^th^ and 15^th^ day after surgery. Functional evaluation was performed by the grasping strength of the right pelvic limb. The right tibialis anterior muscles were evaluated for mass, smaller diameter and cross-sectional area. In the Cryotherapy Injury 8 and the Cryotherapy Injury 15 groups, the hydroxyproline was dosed in the right soles.

**Results:**

In the compression there was a significant difference in the Injury Groups compared with the Control Group (p<0.05). In the smaller diameter, the compression in Control Group was higher than Injury 8 (p=0.0094), Injury 15 (p=0.002) and Cryotherapy Injury 15 (p<0.001) groups. The comparison between groups with euthanasia in the same post-operative period, a significant difference (p=0.0363) was seen in day 8^th^ after surgery, and this result in Cryotherapy Injury Group was greater than Injury Group. In the fiber area, Control Group was also higher than the Injury 8 (p=0.0018), the Injury 15 (p<0.001) and the Cryotherapy Injury 15 (p<0.001). In hydroxyproline, no significant difference was seen between groups.

**Conclusion:**

Nerve damage resulted in decreased muscle strength and trophism, the cryotherapy delayed hypotrophy, but this effect did not persist after cessation of treatment.

## RESUMO

**Objetivo:**

Avaliar os efeitos da compressão nervosa do isquiático direito e da crioterapia no tecido muscular.

**Métodos:**

Foram utilizados 42 ratos Wistar machos, subdivididos nos Grupos Controle, Lesão 3, Lesão 8 e Lesão 15, submetidos a compressão nervosa e eutanasiados, respectivamente, no 3°, 8° e 15° dias pós-operatório; Lesão Crioterapia 3, tratado com crioterapia, por imersão durante 20 minutos, por 1 dia, e eutanasiados no 3° dia pós-operatório; e Lesão Crioterapia 8 e Lesão Crioterapia 15, tratados durante 6 dias e eutanasiados no 8° e 15° dias pós-operatório. A avaliação funcional foi realizada pela força de preensão do membro pélvico direito. Os músculos tibiais anteriores direitos foram avaliados quanto a massa, menor diâmetro e área de secção transversa. Em Lesão Crioterapia 8 e Lesão Crioterapia 15, foi dosada a hidroxiprolina nos sóleos direitos.

**Resultados:**

Na preensão, houve diferença significativa nos Grupos Lesão quando comparados ao Grupo Controle (p<0,05). No menor diâmetro, o Grupo Controle foi maior que Lesão 8 (p=0,0094), Lesão 15 (p = 0,002) e Lesão Crioterapia 15 (p<0,001). Na comparação entre os grupos com eutanásia no mesmo pós-operatório, houve diferença significativa (p=0,0363) no 8° pós-operatório, sendo Lesão Crioterapia maior que Lesão. Na área das fibras, o Grupo Controle também foi maior que Lesão 8 (p=0,0018), Lesão 15 (p<0,001) e Lesão Crioterapia 15 (p<0,001). Na hidroxiprolina, não houve diferença significativa entre os grupos.

**Conclusão:**

A lesão nervosa resultou na diminuição da força e em trofismo muscular, e a crioterapia retardou a hipotrofia, porém este efeito não se manteve após o tratamento cessar.

## INTRODUCTION

Peripheral neuropathies of lower limbs are common injuries often associated with trauma and surgical procedures. These injuries impact significantly the mobility and function of patient, and they are of great relevance for economic issues of the society.^(^ ^1^ ^–^ ^3^ ^)^ The most used model to study this injury is the sciatic nerve compression in rats because, in addition to reduce compressive neuropathy, which is most common in lower limb, it is ease to be accessed surgically.^(^ ^2^ ^,^ ^4^ ^)^

The model of sciatic nerve compression can lead to changes in sensibility, as well as to muscle hypotrophy that is observed in the third day after the surgery or up to around 20 days after the procedure. About 30 days after the procedure, the muscle and nerve recovery spontaneously and it does not allow observation of these effects.^(^ ^5^ ^–^ ^8^ ^)^ However, when muscular hypotrophy is seen, such tissue can be replaced by conjunctive, therefore, mainly formed with collagenous, which result in loss of function, when nerve regeneration does not occur appropriately.^(^ ^9^ ^)^

One the treatment forms for nerve compressions is the use of physical therapy modalities, such as cryotherapy, which is application of cold from 0°C to 18.3°C,^(^ ^10^ ^)^ in which reduce local blood flow, metabolic rate and speed of peripheral nerve conduction. These changes result in a reduction of inflammatory reaction in acute trauma, pain and edema formation.^(^ ^11^ ^)^ The cryotherapy time by immersion technique is around 20 minutes to achieve therapeutic effects because it freezes an larger area than other modalities.^(^ ^10^ ^,^ ^12^ ^–^ ^14^ ^)^ Considering that cryotherapy is ease to access and a low cost therapeutic modality,^(^ ^11^ ^)^ studies are important to verify its efficiency in functional and structural changes of muscular tissue because of nervous injury.

## OBJECTIVE

To evaluate experimental model effects of nerve compression and cryotherapy in muscular tissue of Wistar rats.

## METHODS

We used 42 Wistar male rats with 10 weeks of age obtained from the animal housing of *Universidade Estadual do Oeste do Paraná* (UNIOESTE), Cascavel campus, Brazil. The study started in the beginning of the second semester of 2015 and analyses were carried out up to the second semester of 2016. Animals were housed in polypropylene cages with freely access to water and food, 12 hours light/dark cycles, and controlled temperature 24°C±1°C.

Animals were randomly divided into seven groups (n=6/group): Control Group (C) - animals not submitted to nervous compression and nor to cryotherapy, and euthanized in the 15^th^ day after beginning of the experiment; Injury 3, 8 and 15 group (I3, I8 and I15) submitted to nervous compression and euthanized in 3^rd^, 8^th^ and 15^th^ day after surgery; Cryotherapy Injury 3 (CrioI3) group submitted to nervous compression and cryotherapy during day 1, and euthanized in day 3 after surgery; Cryotherapy Injury 8 and 15 group (CrioI8 and CrioI15) submitted to nerve compression and cryotherapy during 6 days, and euthanized at 8th and 15^th^ day after the surgery.

The project was approved by the Ethical Committee on Animal Research of UNIOESTE, protocol 06611 (June 15, 2014), and it was conducted according to international norms of ethical animal experimentation.

Previous to surgical procedure of sciatic nerve, animals were weighted and anesthetized with ketamine chloride (95mg/kg) and xylazine chlorhydrate (12mg/kg) intraperitoneal. After anesthesia, we verified the consciousness state (lack of motor response to caudal clamping and interdigital folds), subsequently, animals were placed in ventral decubitus position for shaving of surgical site.

After, an incision was carried out to expose the right sciatic nerve, and then nerve compression was conducted using a hemostatic clamping for 30 seconds. Clamping pressure was standardized to all animals, using as reference the second rack tooth.^(^ ^15^ ^)^ Finally, an external suture was performed with Catgut 4.0 thread.

Animals were placed on the right posterior limb immersed in a recipient of 1,440cm^3^ (20cmx12cmx6cm) with water and ice, in a temperature of 5°C±2°C, for 20 minutes.^(^ ^16^ ^)^ The first treatment was conducted in CrioI3, CrioI8 and CrioI15 groups after surgical procedure for nerve compression. From the 3^rd^ day after surgery, cryotherapy was retaken and performed in consecutive days up to the 7^th^ day after the surgery only in CrioI8 and CrioI15 groups.

Animals of I3, I8 and I15 groups were placed within a recipient without water for 20 minutes to adapt to the stress caused by manipulation. Manipulation of I3, I8 and I15 groups corresponded, respectively, with days of cryotherapy treatment of CrioI3, CrioI8 and CrioI15 groups.

For muscle function assessment, we used measurer of grasping strength.^(^ ^17^ ^)^ The test was adapted for grasping strength assessment of right pelvic limb. Animals were hold gently on the dorsum and they were allowed to use right forepaw to grasp a grip connected to a strength transducer. Animals were pulled down firmly until it lose compression, registering in the equipment, at this moment, the maximal strengthen done. In each evaluation, test was repeated three times and the mean value was used. We conducted an adapting and training time with all animals 3 days before to perform the nerve injury.

Test was done before sciatic nerve compression to obtain basal values (AV1) and in the 2^nd^ day after nerve compression to all groups (AV2); posteriorly, a third evaluation was done (AV3), in the 3^rd^ day after surgery for C Groups, I3 and CrioI3; in 8^th^ day after surgery for C, I8 and CrioI8 groups; and 15^th^ day after surgery for C, I15 and CrioI15 groups, which was always done by the same evaluator.

After intervention period for each group, animals were weighted, and anesthetized and euthanized using a guillotine. After that, tibialis anterior muscle was dissected, weighted and fixed in Methacarn for 24 hours, followed by histologic processing of routine for inclusion in paraffin: dehydration in a growing series of alcohol, diaphanization in xylol, infiltration in histologic paraffin, and embedding to obtain crosssectional cuts.

Muscles were cut at 7*μ*m thickness and hematoxylin and eosin (HE) staining. A total of ten fields were photomicrographed aiming 40x and, posteriorly, we analyzed muscles in terms of lower diameter and area per 100 fibers per muscles using the Image-Pro-Plus 6.0 program. In addition, muscles were analyzed in terms of general morphology of the tissue.

Hydroxyproline is the main component of collagenous, and measurement of its levels can be used as an indicator of collagenous compound. The analysis was done in right soleus muscle, in I8, I15, CrioI8 and CrioI15 groups. For this reason, the muscle was collected, frozen in liquid nitrogen and homogenized. The dosage used followed instructions of manufacturer (Sigma-Aldrich®, catalog number MAK008).

### Statistical analysis

Results were expressed by means and standard deviations. The Shapiro-Wilk test was used for data normalization and, subsequently, unidirectional ANOVA statistical test followed by the LSD post-test (*t* test) were used to compare groups at different times. A p value<0.05 was considered significant. For analyses, we used the Bioestat 5.0 program.

## RESULTS

According to grasping strength analysis, we observed for basal values that no significant difference between groups [F(6;35)=1.6; p=0.181)]. However, for AV2, there were differences [F(6;35)=34.7; p<0.001)], and all groups had low strength than C Group. Such fact repeated with AV3, regardless if comparison was 3^rd^ day after surgery [F(2; 15)=6.3; p=0.007)], 8^th^ day after surgery [F(2; 15)=6.5; p=0.009)] or 15^th^ day after surgery [F(2; 15)=6.5; p=0.009)] (Table 1). A reduction in grasping strength after injury, regardless of treatment with cryotherapy, and the strength did not return to basal values.

**Table 1:** Grasping strength of control (C) group, Injury (I3, I8 and I15) group and group treated with cryotherapy (CrioI3, CrioI8, and CrioI15)

Assessment	C	I3	CrioI3	I8	CrioI8	I15	ICrio15
1	72.7±32.5	64.1±36.5	53.9±16.4	41.8±18.9	33.6±10.3	43.2±29.6	57.7±31.5
2	51.3±17.1	6.1±8.7*	0±0*	0±0*	0±0*	0±0*	0±0*
3	66.2±20.7	18.7±29.1*	0±0*	0±0*	0±0*	0±0*	0±0*

* Significant difference compared with Control Group. Results expressed as mean and standard deviation.

For the variable tibialis anterior muscle weight there was significant difference in C Group [F(6; 35)=39.7; p<0.001)], which presented a higher mean than other groups. There was no difference between euthanized animals even among those of the same surgical procedure. A significant difference observed in groups submitted only to nervous injury and mean of I3 was higher than in I8 and I15 groups. The same occurred in the injured group and group treated with cryotherapy, the mean of CrioI3 group was higher than in CrioI8 and CrioI15 group (Table 2).

**Table 2:** Weight of tibialis anterior muscle of Wistar rats

Control	After surgery	Injury	Cryotherapy injury
0.812±0.264	3°	0.584± 0.049*	0.544±0.032*
	8°	0.397±0.033* †	0.442±0.043* ‡
	15°	0.359±0.012* †	0.347 ± 0.014* ‡

* Difference between Control Group;

† difference in relation to injury 3 group;

‡ difference in relation to cryotherapy injury 8 group. Results reported as standard deviation and means.

In morphometric measurement for analysis of lower diameter of tibialis anterior muscle fiber, there was significant difference [F(6;35)=54.0; p=0.001]; and the C Group was higher than in I8 (p=0.009), I15 (p=0.002) and CrioI15 (p< 0.001) groups. Comparison between euthanized groups after surgery showed significant difference (p=0.036) in 8th day after the surgery, and CrioI group that had greater difference than in I group (Table 3).

**Table 3:** Diameter of tibialis anterior muscle fiber of Wistar rats

Control	After surgery	Injury	Cryotherapy injury
37.61±4.58	3°	34.71±2.72	37.08±2.54
	8°	31.85±3.16*	36.07±4.01†
	15°	30.33±2.42*	30.08±3.44*

* Difference in relation to Control Group;

† difference in relation to Injury 8 group. Results are reported as means and standard deviations.

In the analysis of tibialis anterior muscle fibers, there was also a significant difference [F(6;35)=83.7; p<0.001], and C Group was greater than in I8 (p=0.002), I15 (p<0.001), and CrioI15 (p<0.001). There was no difference between euthanized groups in the same surgery (Table 4).

**Table 4:** Mean and standard deviation of tibialis anterior muscle fibers of Wistar rats

Control	After surgery	Injury	Cryotherapy injury
1,726±400	3°	1,465±188	1,732 ± 245
	8°	1,190±170*	1,469±332
	15°	1,005±163*	1,095±161*

* Difference between Control Group. Results are reported as mean and standard deviation.

**Figure 1:**
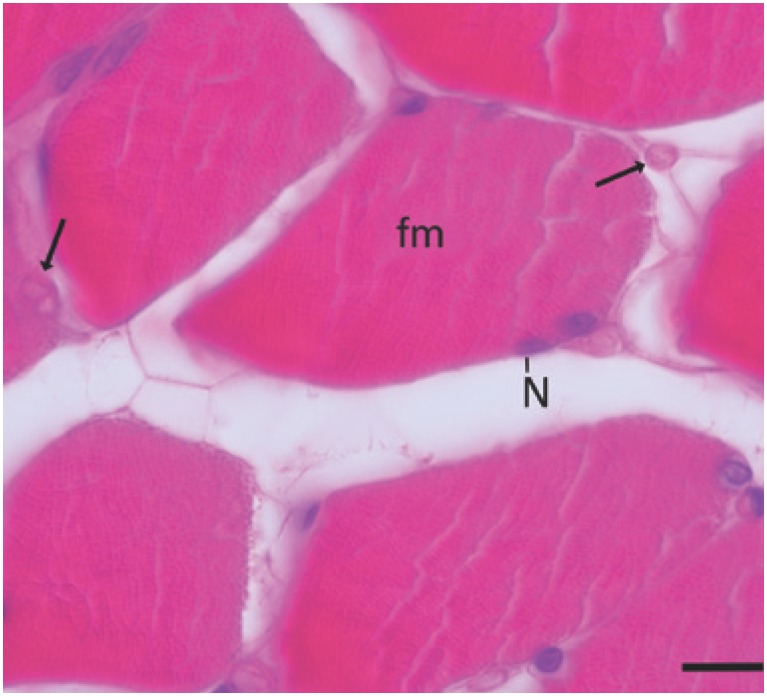
Photomicrography of tibialis anterior muscle of Wistar rates of Control Group, cross-sectional cut, hematoxylin and eosin staining, 1000x. 10μm bar. Muscle fibers (mf) with polygonal format, peripheric nucleus (N) and normal fascicular pattern. Capillary vessel (arrow) in endomysial conjunctive

**Figure 2:**
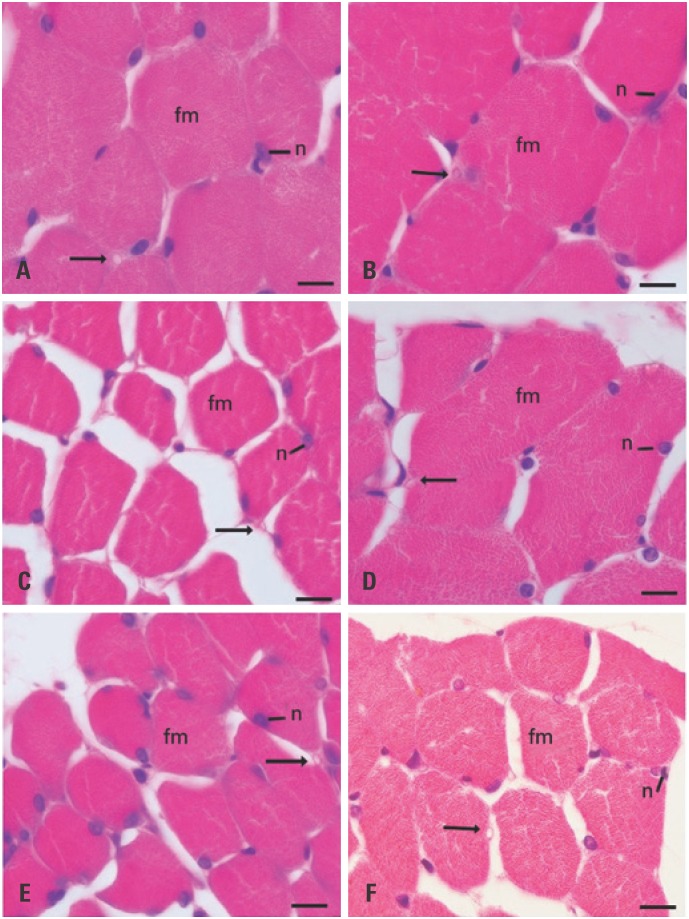
Photomicrography of tibialis anterior muscle of Wistar rats, crosssectional cut, hematoxylin and eosin staining. 1,000 x. 10*μ*m bar. Groups: (A) Injury 3, (B) Cryotherapy Injury 3, (C) Injury 8, (D) Cryotherapy Injury 8, (E) Injury 15, (F) Cryotherapy Injury 15. In Injury 3 group, Cryotherapy Injury 3 and Cryotherapy Injury 8 group, we could observe normal fascicular pattern of the tissue, muscle fibers with polygonal format, peripheral nucleus and complete capillary vessels (arrows) in conjunctive (A, B and D). Despite Injury 8, Injury 15, and Cryotherapy Injury 15 group had large amount of fiber of polymorphic muscles with reduce of its size, they maintained the peripheral position of cell nucleus (n) (C, E and F)

Muscular tissue in C Group had normal fascicular standard with high cellularity and richly supplied by blood veins in endomysium, perimysium and epimysium conjunctive. Muscle fibers presented a polygonal format, approximately hexagonal, with an individual cells modeled one to another, peripheral nucleus and complete capillary vessel in endomysium (Figure 1). These same morphological characteristics were observed in I3, CrioI3 and CrioI8 (Figure 2A; 2B and 2D) group. The I8, I15 and CrioI15 groups present large amount of polymorphic muscles with apparent reduction in size, however, there were maintenance peripheral positioning of cell nucleus (Figure 2C; 2E and 2F).

In the hydroxyprolyne analysis, there was no significant difference [F(3;15)=1.1; p-0.379)] between I and euthanized CrioI group in 8^th^ day and 15^th^ day after surgery (Table 5).

**Table 5:** Hydroxyproline dosage of soleus muscle

After surgery	Injury	Cryotherapy injury
8°	0.076±0.03*μ*g/uL	0.12±0.07*μ*g/*μ*L
15°	0.11±0.07*μ*g/*μ*L	0.06±0.04*μ*g/*μ*L

## DISCUSSION

Peripheral nervous injuries directly affect the muscle tissue because they interrupt the neuromuscular communication^(^ ^18^ ^)^ and result in loss of function of innervated organ,^(^ ^19^ ^)^ and reduction of cross-sectional section and muscle diameter of muscle fibers.^(^ ^20^ ^)^ In our study, changes in muscle structure, such as weight reduction, diameter and muscle area were observed in injured groups since 8^th^ day after surgery, but, when cryotherapy was done, these changes were observed only in 15^th^ day after surgery, therefore, physiotherapeutic modality reduced speed of muscle atrophy.

Few hours after nervous injury, there a release of inflammatory mediators by mastocyte such as histamine and chemokine that contributed for recruiting other inflammatory cells and, consequently, for development of hyperalgesia.^(^ ^7^ ^)^ Such hyperalgesia and change of muscle innervation resulted in a reduction of use of affected limb, generating muscle atrophy. Cryotherapy can result in reduction of nerve conductibility and, therefore, anti-inflammatory effects produced with decrease of release of pain factors,^(^ ^11^ ^)^ which could avoid disuse of animal limb, and prevent muscle hypotrophy.

Euthanize proposed in our study occurred in 3rd day and 15th day after the surgery because 3 days after peripheral nervous injury, no muscle hypotrophy characteristics were observed;^(^ ^5^ ^)^ and within 2 weeks of injury, Wallerian degeneration is well documented,^(^ ^15^ ^)^ *i.e.,* the occurrence of change with visits to reduce hypotrophy can be computed by the cryotherapy.

After finishing cryotherapy, we observed muscle atrophy clinical presentation, possibly because of the non-use of pelvic limb and hyperalgesia that, for the proposed model, reached the peak around 14^th^ day after the surgery.^(^ ^7^ ^)^ We understand that, for the cryotherapy protocol discontinuation, the maintenance of the muscle tropism could occur. For this reason, we suggest new studies with the use of more extensive protocol using cryotherapy.

Cryotherapy has been reported as way to preserve neuronal integrity after injury, such as presented in a study by Cruz et al.,^(^ ^21^ ^)^ that used such modality associated with alkalinization of tissue in animals submitted to injury by prolonged ischemia. This therapeutic association was efficient to preserve neurons in the dorsal root ganglion. Kawamura et al.,^(^ ^22^ ^)^ also evaluated cryotherapy effects in animals submitted to ischemia, and they observe that cryotherapy, applied during the ischemia period, was efficient in neuronal protection - a fact that did not occur when cryotherapy was used during reperfusion.

In addition to changes in structure, the peripheral nervous injury results in functional change, which is observed by reduction of strength, a result seen in our study. This reduction could occur due to changes in structure of neural and/or muscle.^(^ ^9^ ^)^ Probably, this reduction of strength in our study was due to neural changes, especially because after traumatic nervous injury a change occurred in electromyography and reduction of recruiting of health motor units.^(^ ^23^ ^)^ We highlight that reduction of strength was associated with muscle structure because it occurred in all groups in the same period, *i.e.,* after the 2^nd^ day of surgery, while changes in muscle structure began in different times, depending of the use of cryotherapy.

Although regenerative process was expected, the period of the disease implicates in functional and structural losses, justifying the partial denervation. Such functional losses are characterized, mainly, for the reduction of muscle tonus and, consequently, capacity to generate strength.^(^ ^24^ ^)^ After nervous injury, in addition to described morphological changes, we also observed changes in muscle excitability^(^ ^25^ ^)^ and increase in density of conjunctive tissue,^(^ ^24^ ^)^ although in experimental models this fact not always occur.^(^ ^20^ ^)^

Nervous injury led to interruption of neuromuscular communication.^(^ ^26^ ^,^ ^27^ ^)^ In our study, lack of stimulus changed the morphology of tibialis anterior muscle of animals in the Injured Group, which revealed typical characteristics of muscle injury caused by denervation with polymorphic muscle fibers with apparent reduction of its size, therefore, compromising function of tissue.^(^ ^28^ ^)^

Because of change in 8^th^ day after surgery, a complementary analysis of conjunctive tissue was conducted by quantification of hydroxiplolin only in injured and treated groups at 8^th^ day and 15^th^ day after surgery in soleus muscl. However, no significant difference was seen. Although these muscles have different structure and function, both receive innervation of tibialis nerve, which is branch of sciatic nerve.^(^ ^4^ ^)^ The hydroxyproline occurs as a biological marker of catabolism/anabolism of collagenous of locomotive apparatus.^(^ ^29^ ^)^ Still, in the literature, a consensus exist related with identification of concentration of collagenous related with biochemistry components, in which, they are defined as determinant for regeneration of harms by increase of molecular bioactivity.^(^ ^30^ ^)^

No difference was observed in collagen deposition between treated group and injured only group, but we highlight that, because of technical difficulties, this assessment was not performed in the C Group. Future studies should perform such comparison.

Another limitation of this study was the lack of evaluation of inflammatory markers, both systemic and local markers, in muscle tissue mediated by neurogenic inflammation process.^(^ ^31^ ^,^ ^32^ ^)^ Future studies should explore such evaluations and include human subjects with acute and short-time peripheral nerve injuries.

## CONCLUSION

Nerve injury resulted in a reduction of strength and muscle trophism (weight, diameter and area). The cryotherapy caused a delaying in hypotrophy during its application, however, this effect did not last after treatment was ceased.
